# Etymologia: **Calicivirus** [kә-lis′ ǐ-vi′′rәs]

**DOI:** 10.3201/eid1512.999999

**Published:** 2009-12

The name of these members of the Caliciviridae family of nonenveloped RNA viruses reflects their structure. They are icosahedral with 32 typical surface depressions that are sometimes described as hollows or cups (from Latin *calyx,* meaning cup). Feline calicivirus, a member of the genus *Vesivirus*, causes respiratory disease in cats. Members of other genera, *Norovirus* and *Sapovirus*, cause gastrointestinal disease in humans.

